# Highly efficient production of transfructosylating enzymes using low-cost sugarcane molasses by *A. pullulans* FRR 5284

**DOI:** 10.1186/s40643-021-00399-x

**Published:** 2021-06-11

**Authors:** Most Sheauly Khatun, Morteza Hassanpour, Mark D. Harrison, Robert E. Speight, Ian M. O’Hara, Zhanying Zhang

**Affiliations:** 1 Centre for Agriculture and the Bioeconomy, Faculty of Science, Queensland University of Technology, Brisbane, QLD 4000 Australia; 2 School of Mechanical, Medical and Process Engineering, Faculty of Engineering, Queensland University of Technology, Brisbane, QLD 4000 Australia; 3 School of Biology and Environmental Science, Faculty of Science, Queensland University of Technology, Brisbane, QLD 4000 Australia; 4 Centre of Excellence in Synthetic Biology, Queensland University of Technology, Brisbane, QLD 4000 Australia

**Keywords:** Molasses, *Aureobasidium*, Transfructosylation, Fructooligosaccharides, Nitrogen, Scale-up

## Abstract

Fructooligosaccharides (FOS) are a type of important prebiotics and produced by transfructosylating enzymes. In this study, sugarcane molasses was used as the substrate for production of transfructosylating enzymes by *Aureobasidium pullulans* FRR 5284. NaNO_3_ was a superior nitrogen source to yeast extract for production of transfructosylating enzymes by *A. pullulans* FRR 5284 and decreasing the ratio of NaNO_3_ to yeast extract nitrogen from 1:0 to 1:1 resulted in the reduction of the total transfructosylating activity from 109.8 U/mL to 82.5 U/mL. The addition of only 4.4 g/L NaNO_3_ into molasses-based medium containing 100 g/L mono- and di-saccharides resulted in total transfructosylating activity of 123.8 U/mL. Scale-up of the *A. pullulans* FRR 5284 transfructosylating enzyme production process from shake flasks to 1 L bioreactors improved the enzyme activity and productivity to 171.7 U/mL and 3.58 U/mL/h, 39% and 108% higher than those achieved from shake flasks, respectively. Sucrose (500 g/L) was used as a substrate for extracellular, intracellular, and total *A. pullulans* FRR 5284 transfructosylating enzymes, with a maximum yield of 61%. Intracellular, extracellular, and total *A. pullulans* FRR 5284 transfructosylating enzymes from different production systems resulted in different FOS profiles, indicating that FOS profiles can be controlled by adjusting intracellular and extracellular enzyme ratios and, hence prebiotic activity.

**Supplementary Information:**

The online version contains supplementary material available at 10.1186/s40643-021-00399-x.

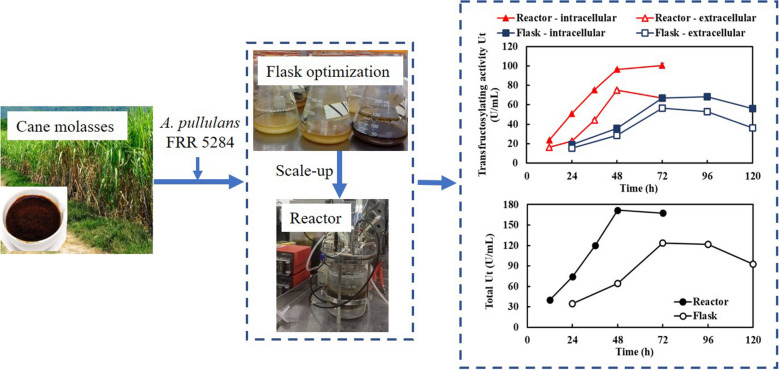

## Introduction

*Aureobasidium* is a genus of microorganisms with substantial potential as a source of transfructosylating enzymes *β*-d-fructofuranosidase (FFase, EC 3.2.1.26) and fructosyltransferase (FTase, EC 2.4.1.9). These enzymes convert sucrose and inulin to fructooligosaccharides (FOS), a class of oligosaccharides used as food and feed prebiotics (Bali et al. 2015; Flores-Maltos et al. 2016). The primary FOS synthesized by microbial transfructosylating enzymes are 1-kestose (GF_2_), nystose (GF_3_), and 1,1,1-kestopentaose (GF_4_). Microbial transfructosylating enzymes are often produced using sucrose as a carbon source (Bali et al. 2015; Flores-Maltos et al. 2016). High-purity sucrose is commercially produced from sugarcane and sugar beet juice after removal of reducing sugars (glucose and fructose) and other impurities (e.g., protein, minerals, and colorants). Molasses is a sucrose-rich, viscous liquid by-product of high-purity sucrose manufacture from sugarcane juice and is generated after during sucrose crystallization. Although high-purity sucrose is the preferred carbon source for production of high-purity FOS for human consumption, the microbial production of transfructosylating enzymes does not require high-purity sucrose as a carbon source.

Nitrogen is an essential nutrient for microbial growth and can be supplied to microorganisms in inorganic (e.g., NaNO_3_) or organic (e.g., yeast extract) compounds. The effect of nitrogen source and concentration on transfructosylating enzyme production by microorganisms, such as *Aspergillus* strains, has been evaluated (Ashok Kumar et al. 2001; Dorta et al. 2006). Further, optimal nitrogen concentration for microbial transfructosylating enzyme production is affected by the concentration of other nutrients in the fermentation media, including phosphorus and magnesium (Ashok Kumar et al. 2001; Vandáková et al. 2004). Despite the importance of nitrogen source, concentration, and interaction with other micronutrients on microbial enzyme production, research on the effect of these factors on transfructosylating enzyme production by *Aureobasidium* strains is limited. *A. pullulans* CCY 27-1-94 FFase production was maximal in in synthetic sucrose medium containing 10 g/L yeast extract and 10 g/L NaNO_3_ (Vandáková et al. 2004). Subsequent studies demonstrated that production of transfructosylating enzymes by *Aureobasidium* strains could be maximized by the inclusion of different ratios of yeast extract and NaNO_3_ at concentrations of 10–20 g/L in pure sucrose fermentation media (Aung et al. 2019; Jung et al. 2011; Salinas and Perotti 2009; Zhang et al. 2019).

Molasses contains 30–60% sucrose, 6–12% glucose, and 6–12% fructose by weight (Bortolussi and O’Neill 2006; Dorta et al. 2006; Zhang et al. 2019). Molasses also contains proteins, minerals (such as calcium, potassium, magnesium and phosphorus), and vitamins, which are either derived from harvested sugarcane or from additives used in sugarcane juice clarification (such as lime and phosphoric acid) (Doherty 2011; Mee et al. 1979; Thai et al. 2012). Molasses is often directly used as a feed supplement or fermented to bioproducts such as ethanol and microbial oils (Bento et al. 2020; Mordenti et al. 2021). Molasses has also been evaluated as a carbon source for microbial production of transfructosylating enzymes, but such studies were limited to *Aspergillus* strains (Dorta et al. 2006; Xie et al. 2017). The highest transfructosylating activity produced by *Aspergillus* was in molasses-based fermentation media containing 15 g/L yeast powder (Dorta et al. 2006). While molasses has been used as a carbon source for the production of FOS by *Aureobasidium* strains, the production of transfructosylating enzymes by these strains was not evaluated (Khatun et al. 2020; Shin et al. 2004; Zhang et al. 2019). Given the composition of molasses, the requirement for additional micronutrients in molasses-based microbial fermentation media for the production of transfructosylating enzymes should be lower than sucrose-based fermentation media, thereby significantly reducing the cost of transfructosylating enzyme production.

Transfructosylating enzymes by *A. pullulans* form a relatively small protein family consisting of at least five members and each member has different transfructosylating and hydrolytic activities (Yoshikawa et al. 2006). Individual transfructosylating enzymes accumulate inside the cell or are secreted into the fermentation media; as a result, the mixtures of intracellular and extracellular transfructosylating enzymes in a given microbial strain differ in their total transfructosylating and hydrolytic activities. As a result, it is likely that FOS produced using intracellular or extracellular transfructosylating enzymes will have different ratios of GF_2_, GF_3_, and GF_4_. Therefore, the use of combinations of intracellular and extracellular transfructosylating enzymes will allow FOS profiles (and therefore prebiotic activity) to be optimized for specific food and feed applications.

In the present study, molasses was evaluated as a relatively low-cost carbon source for production of transfructosylating enzymes by a recently identified, elite *A. pullulans* strain FRR 5284. The effect of exogenous nitrogen source, total concentration of exogenous nitrogen, and the addition of phosphorus to the fermentation media on transfructosylating activity was investigated in shake-flask cultures. Fermentation of *A. pullulans* FRR 5284 under optimal conditions for transfructosylating enzyme production was scaled up to 1 L using a stirred tank bioreactor, and FOS production using intracellular, extracellular, and mixtures of intracellular and extracellular transfructosylating enzymes produced in shake-flask and bioreactor fermentations was compared. The results of this study provide key information for the optimization and scale-up of transfructosylating enzyme production by *A. pullulans* using molasses as a carbon source and identify a simple strategy to control FOS profile during synthesis from sucrose by *A. pullulans* transfructosylating enzymes.

## Materials and methods

### Materials

Sugarcane molasses was supplied by the Racecourse Sugar Mill in Mackay, Australia. The molasses contained 41.7% sucrose, 7.4% glucose and 5.9% fructose by weight, as determined by high performance chromatography (HPLC) analysis (details of the analysis are presented below). The molasses also contained 4.3 g/kg total nitrogen, 1.8 g/kg phosphorus, 2.8 g/kg magnesium, 9.5 g/kg potassium, and 2.3 g/kg calcium, as determined using inductively coupled plasma optical emission spectrometry (see Additional file 1). Sucrose, glucose, fructose, GF_2_, and GF_3_ were purchased from Sigma-Aldrich (US). GF_4_ was obtained from Megazyme (Ireland). All other chemicals were purchased from Sigma-Aldrich (US), except for yeast extract (Merck, Germany). All the chemicals used in this study were analytical grade or above.

*Aureobasidium pullulans* FRR 5284 was purchased from the Australian CSIRO Fungal Culture Collection. The strain was identified as a highly efficient transfructosylating enzyme producer by the author in a recent study (Khatun et al. 2020) and was stored in 30% (v/v) glycerol in water at − 80 °C.

### Preparation of *A. pullulans* FRR 5284 inoculum

Inoculum medium was prepared as described previously (Khatun et al. 2020). Glycerol stocks of *A. pullulans* FRR 5284 were streaked onto YPD agar plates and incubated at 28 °C for 2 days to generate spores. Sporulated *A. pullulans* FRR 5284 was stored on agar plates at 4 °C for short-term use (i.e., within 30 days).

Inoculum medium contained 20 g/L glucose, 20 g/L peptone, and 10 g/L yeast extract. Inoculum medium pH was adjusted to 5.5 by the addition of 2 M HCl or 2 M NaOH and then sterilized at 121 °C for 15 min. Single colonies of *A. pullulans* FRR 5284 were transferred via sterile loop into 10 mL of inoculum medium in 50 mL centrifuge tubes and grown for 2 days at 28 °C and 180 rpm. *A. pullulans* FRR 5284 cells were harvested by centrifugation at 4000 rpm for 10 min. The supernatant was discarded, and the cells were resuspended in two aliquots (10 mL) of sterile water. The cell suspensions were centrifuged at 4000 rpm for 10 min and the washed cells were resuspended in water at a loading of 2–3 g/L to serves as the inoculum for shake-flask fermentation.

*Aureobasidium pullulans* FRR 5284 inoculum for bioreactor fermentation was prepared in a two-step process. The first step was similar to that described for shake-flask fermentation with the following modification: after washing and re-suspension in sterile water, 10 mL of cell suspension was transferred into 250-mL shake flasks containing 90 mL diluted molasses (total sugar = 20 g/L sucrose equivalents), 20 g/L peptone, and 10 g/L yeast extract, and grown for 48 h at 28 °C and 180 rpm. *A. pullulans* FRR 5284 cells were harvested, washed, and resuspended in water to the same loading as descried previously.

### Production of transfructosylating enzymes by *A. pullulans* FRR 5284 in shake flasks

#### NaNO_3_ and yeast extract combination

Sugarcane molasses was used as a low-cost sucrose source for production of transfructosylating enzymes by *A. pullulans* FRR 5284 at a total sugar concentration of 100 g/L (sucrose equivalent). Two exogenous nitrogen sources (NaNO_3_ and yeast extract) were added to the molasses medium at NaNO_3_ nitrogen (N1)/yeast extract nitrogen (N2) ratios of 0:0 (control, no exogenous nitrogen), 1:0, 1:1, and 3:1 at a total nitrogen concentration of 2.48 g/L (equivalent to 15.0 g/L NaNO_3_). The total nitrogen concentration in the fermentation media included the 0.92 g/L nitrogen in molasses (equivalent to 5.6 g/L NaNO_3_). Fermentation media were supplemented with an exogenous phosphorous source (Na_2_HPO_4_) to a total phosphorus concentration of 0.87 g/L (equivalent to 4.0 g/L Na_2_HPO_4_). The total phosphorous concentration in the fermentation media included the 0.40 g/L phosphorous in molasses (equivalent to 1.8 g/L Na_2_HPO_4_). Other exogenous nutrients, such as magnesium and potassium salts, were not added to the fermentation medium as these nutrients were already at relatively high concentrations (3.0 g/L MgSO_4_ equivalent, 4.1 g/L KCl equivalent) in molasses-based media at 100 g/L total sugar concentration (sucrose equivalents).

*Aureobasidium pullulans* FRR 5284 fermentations were performed in 250-mL shake flasks containing 50 mL fermentation medium in triplicate. Aliquots (5 mL) of inoculum were transferred into the shake flasks and grown for 120 h at 28 °C and 180 rpm. Homogeneous sub-samples (5 mL) were collected after 24, 48, 72, 96, and 120 h. *A. pullulans* FRR 5284 cells were harvested by centrifugation at 4000×*g* for 15 min. Sub-samples of the supernatants were collected for identification and quantification of residual sugars and FOS by HPLC. The remainder of the supernatants were stored at − 20 °C for measurement of extracellular enzyme activity. *A. pullulans* FRR 5284 cells were washed with twice with deionized water (10 mL) and freeze-dried for 48 h. Freeze-dried *A. pullulans* FRR 5284 cells were stored at 4 °C for measurement of intracellular enzyme activity.

#### Exogenous NaNO_3_ and Na_2_HPO_4_

Based on the results of the experiments to identify the optimal fermentation media composition for transfructosylating enzyme production by *A. pullulans* FRR 5284 (as per the previous section), NaNO_3_ was selected as the sole nitrogen source for subsequent fermentation experiments with Na_2_HPO_4_ as the exogenous phosphorous source. Three total nitrogen concentrations in the fermentation media were evaluated [0.92 (no exogenous nitrogen), 1.65, and 2.48 g/L] in combination with three total phosphorus concentrations (0.40, 0.65, and 0.87 g/L), which corresponded to the equivalent of 5.6, 10.0, and 15.0 g/L NaNO_3_ and 1.8, 3.0 and 4.0 g/L Na_2_HPO_4_, respectively. The remaining fermentation conditions and the experimental procedure were as described in the previous section.

### Production of transfructosylating enzymes by *A. pullulans* FRR 5284 in 1 L bioreactors

*Aureobasidium pullulans* FRR 5284 fermentation was undertaken in a 1 L bioreactor (Sartorius Biostat Q-Plus) with a 500 mL working volume. The prepared seed culture was inoculated to the reactor medium to achieve an initial cell concentration of 2–3 g/L. The dissolved oxygen level was maintained above 20% by controlling the agitation speed from 350 to 500 rpm and the air flow rate from 0.5 to 0.9 vvm. *A. pullulans* FRR 5284 fermentation was allowed to proceed for 72 h at 28 °C; the fermentation time was selected based on the capacity of *A. pullulans* FRR 5284 to almost completely consume fermentable carbon in 72 h in preliminary experiments (*data not shown*). Homogenous sub-samples (15 mL) were collected after 12, 24, 36, 48, and 72 h, respectively. The sub-samples were clarified by centrifugation at 4000 rpm for 15 min, and the supernatant and cells were processed for storage as described in previous section. All bioreactor-scale fermentations were conducted in duplicate.

### Enzyme activity assays

The transfructosylating and FOS hydrolysis activities in washed and freeze-dried *A. pullulans* FRR 5284 cells (intracellular) and fermentation media (extracellular) were measured as described previously (Khatun et al. 2020; Zhang et al. 2016). Intracellular enzyme activities were measured as follows: freeze-dried cells were resuspended in 5 mL of 50% (w/v) sucrose in 50 mM sodium acetate buffer, pH 5.5, to a final loading of 0.5 g/L. Extracellular enzyme activities were measured using the same sucrose solution using 0.1 mL samples of fermentation medium. Enzyme assays were undertaken at 55 °C with stirring at 100 rpm for 1 h, followed by boiling the reaction mixture for 15 min to stop the reactions. Residual sucrose, glucose, fructose, and FOS were identified and quantified using HPLC.

### FOS production from sucrose

FOS production was undertaken using washed *A. pullulans* FRR 5284 cells, clarified fermentation medium, and unclarified fermentation medium (containing both *A. pullulans* FRR 5284 cells and fermentation medium). Samples of cells and clarified and unclarified fermentation medium for FOS production were collected after 72-h fermentation in shake flasks and 48-h fermentation in the bioreactor [i.e., the timepoints with the highest transfructosylating activity (U/mL) in each fermentation system]. In each FOS production assay, the total transfructosylating activity was 20 U/mL of assay solution (i.e., equivalent to 4.34 mg cells/mL reaction solution, 0.32 mL clarified fermentation medium/mL reaction solution, and 0.16 mL unclarified fermentation medium/mL reaction solution, respectively).

FOS production was performed at 55 °C and pH 5.5 with a stirring speed of 100 rpm for 12 h using pure sucrose (initial concentration = 500 g/L). Sub-samples (0.5 mL) were collected after 1, 3, 6, and 12 h. FOS production assays were conducted in triplicate.

### Identification and quantification of mono- and di-saccharides, and FOS

Sucrose, fructose, glucose, and FOS in fermentation medium were identified and quantified using the HPLC methods described previously study (Khatun et al. 2020). Sucrose concentrations in the FOS production assay were identified and quantified using the same method.

### Calculations of enzyme activities and FOS yields

The specific intracellular and extracellular transfructosylating activities were expressed on a per mg cells (dry weight) or per mL supernatant basis, as previously described (Khatun et al. 2020; Zhang et al. 2016). Intracellular and extracellular transfructosylating activities were also expressed per unit of culture volume (U/mL). The yields of individual FOS and total FOS were calculated by comparison to the initial sucrose concentration in the FOS production assay.

### Statistical analysis

All the experiments in this study were undertaken in triplicate unless otherwise stated. The mean values were reported with standard deviations. Statistical analysis was performed using the Student’s *t*-test and *p* < 0.05 was considered significant.

## Results and discussion

### Effect of exogenous nitrogen and phosphorous on transfructosylating enzyme production by *A. pullulans* FRR 5284 in shake flasks

#### Effect of inorganic/organic nitrogen ratio

NaNO_3_ and yeast extract are two commonly used nitrogen sources for transfructosylating enzyme production by *Aureobasidium* strains. NaNO_3_ has a selling price of US$300–$700/tonne while the price of yeast extract based on per mass unit of nitrogen is several times higher than NaNO_3_ according to some e-commerce websites (e.g., Alibaba). These two nitrogen sources have been typically used together at relatively high concentrations (10–20 g/L) in sucrose-based fermentation media with the addition of other nutrients, such as phosphorous, magnesium and potassium (Aung et al. 2019; Jung et al. 2011; Salinas and Perotti 2009; Yoshikawa et al. 2006; Zhang et al. 2019). From the economic point of view, NaNO_3_ is a preferred nitrogen source compared to yeast extract. Molasses-based fermentation media were used to produce *A. pullulans* FRR 5284 in the present study and, given that molasses is relatively rich in micronutrients, only exogenous nitrogen and phosphorus were added to molasses-based fermentation media.

**Fig. 1:**
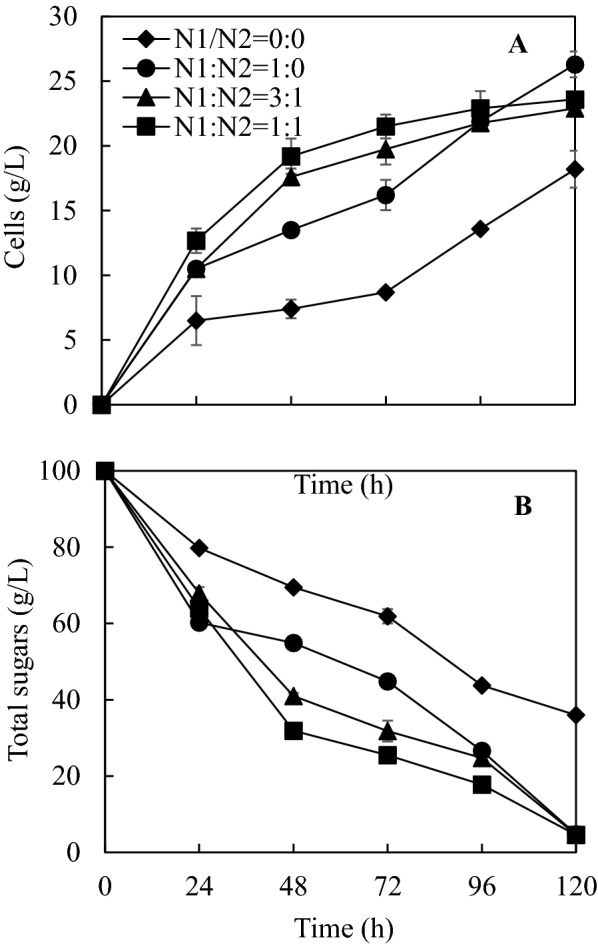
Effect of nitrogen source ratio on cell growth (**A**) and sugar consumption (**B**) in shake-flask cultivations. N1—NaNO_3_ nitrogen, N2—yeast extract nitrogen; total levels of N and P shown in Table 1

The effects of molar ratios of nitrogen from inorganic and organic sources (NaNO_3_ and yeast extract) on transfructosylating enzyme activity by *A. pullulans* FRR 5284 in the presence of a total of 0.87 g/L phosphorous (4.0 g/L Na_2_HPO_4_ equivalent) in molasses-based fermentation media were investigated (Fig. 1). The addition of exogenous nitrogen to the fermentation medium increased *A. pullulans* FRR 5284 biomass production, sugar consumption, and reducing the NaNO_3_ nitrogen/yeast extract-nitrogen (N1/N2) ratio at the same total nitrogen concentration led to rapid cell biomass production and sugar consumption, particularly in the first 96 h of fermentation. In many previous studies, yeast extract is often combined with NaNO_3_ for transfructosylating enzyme production by *Aureobasidium* strains in synthetic sucrose media. Yeast extract not only contains organics nitrogen, but also other nutrients such as vitamins and trace metals, which favor the growth of microorganisms. However, higher concentration of organic nitrogen may lead to over-growth of microorganisms but reduced production of target products, which was observed in this study. A previous study reported that a NaNO_3_/yeast extract mass ratio of 1:1 (or N1/N2 =  ~ 1.6/1.0) was reported to be the optimal for producing transfructosylating enzymes by *A. pullulans* CCY 27-1-94 (Vandáková et al. 2004). In contrast, molasses contains protein and amino acids, which already supplies organic nitrogen (Mee et al. 1979). Therefore, exogenous organic nitrogen may not be necessary for some microbial fermentation processes, depending on the microorganisms and target products.

**Fig. 2:**
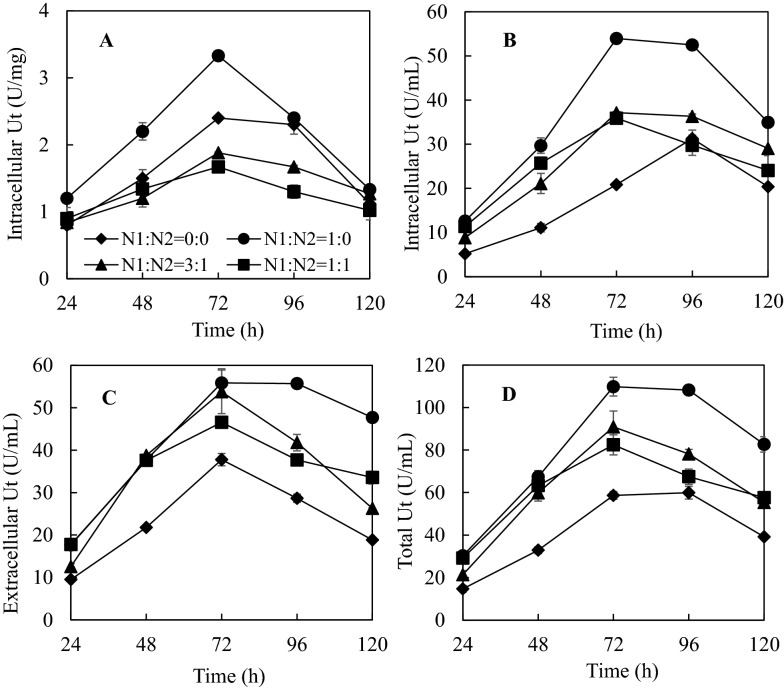
Effect of nitrogen source ratio on specific intracellular transfructosylating activity (**A**), and intracellular (**B**), extracellular (**C**), and total (**D**) enzyme activities based on per unit of volume. N1—NaNO3 nitrogen, N2—yeast extract nitrogen; total levels of N and P shown in Table 1

Intracellular, extracellular, and total transfructosylating enzyme activity from *A. pullulans* FRR 5284 cells grown in different molar ratios of nitrogen from inorganic and organic sources (NaNO_3_ and yeast extract) in the presence of a total of 0.87 g/L phosphorous (4.0 g/L Na_2_HPO_4_ equivalent) were measured (Fig. 2). Intracellular (Fig. 2A) and extracellular (Fig. 2C) specific transfructosylating enzyme activities increased to a maximum at 72 h in fermentation media without exogenous nitrogen. The addition of exogenous nitrogen at a ratio of inorganic to organic nitrogen of 3:1 did not significantly increase intracellular specific transfructosylating enzyme activity (Fig. 2A), but did increase intracellular specific transfructosylating enzyme activity at all other ratios of inorganic to organic nitrogen. The addition of exogenous nitrogen increased extracellular specific transfructosylating enzyme activity irrespective of the inorganic to organic nitrogen ratio (Fig. 2C). Volumetric intracellular transfructosylating enzyme activity reached a maximum at 96 h in the absence of exogenous nitrogen (Fig. 2B); in contrast, the addition of exogenous nitrogen to the culture medium resulted in maximum volumetric intracellular transfructosylating enzyme activity after only 72 h, irrespective of the ratio of inorganic to organic nitrogen. Total transfructosylating enzyme activity in the fermentations (Fig. 2D), a combination of the biomass yield (Fig. 1) and the intracellular (Fig. 2A) and extracellular (Fig. 2C) specific transfructosylating enzyme activities, reached a maximum at 72 h, irrespective of the addition of exogenous nitrogen or the ratio of inorganic and organic nitrogen in the exogenous nitrogen. Table 1 provides a summary of the different fermentation media compositions and the different transfructosylating enzyme activities after 72-h fermentation. Overall, the results indicate that the addition of exogenous nitrogen enhanced both intracellular and extracellular *A. pullulans* FRR 5284 transfructosylating enzyme activity, and that increasing the proportion of exogenous nitrogen supplied by yeast extract reduced total transfructosylating enzyme activity despite increasing *A. pullulans* FRR 5284 biomass yields and sugar consumption.

**Table 1:** Transfructosylating activities of intracellular, extracellular, and total enzymes after 72 h of shake-flask cultivation with two exogenous nitrogen sources at different ratios

N1/N2^a^	Total (g/L)		Salt equivalent (g/L)		Cells (g/L)	Transfructosylating activity			
	N	P	NaNO_3_	Na_2_HPO_4_		Intracellular (U/mg)	Intracellular (U/mL)	Extracellular (U/mL)	Total (U/mL)
0:0^b^	0.92	0.87	5.6	4	8.7 ± 0.2	2.4 ± 0.0	20.9 ± 0.3	37.8 ± 1.5	58.7 ± 1.8
1:0	2.48	0.87	15	4	16.2 ± 1.2	3.3 ± 0.0	53.9 ± 1.1	55.9 ± 3.3	109.8 ± 4.4
3:1	2.48	0.87	15	4	19.8 ± 1.2	1.9 ± 0.0	37.1 ± 2.3	53.8 ± 5.1	90.9 ± 7.4
1:1	2.48	0.87	15	4	21.5 ± 0.9	1.7 ± 0.0	35.9 ± 0.9	46.6 ± 0.9	82.5 ± 4.7

^a^N1—NaNO_3_ nitrogen, N2—yeast extract nitrogen

^b^Exogenous nitrogen sources were not used

#### Effect of exogenous NaNO_3_ and Na_2_HPO_4_

The addition of exogenous inorganic nitrogen (NaNO_3_) to a total nitrogen content of 2.48 g/L in molasses-based fermentation media for *A. pullulans* FRR 5284 resulted in maximum total volumetric transfructosylating enzyme activity after 72 h. To further optimize the concentrations of exogenous nutrients in molasses-based media on transfructosylating enzyme activity, the effects of different combinations of exogenous nitrogen and phosphorous were evaluated. As shown in Additional file 1: Fig. S1, in general high levels of nitrogen and phosphorous led to more rapid cell biomass production and sugar consumption which was a similar observation to that by previous investigation (Hayashi et al. 1992; Vandáková et al. 2004). At the highest levels of nitrogen and phosphorous, residual sugars were less than 5.0 g/L compared to approximately 47 g/L sugars remaining in the molasses-based fermentation medium without addition of exogenous nutrients.

Table 2 summarizes the total transfructosylating activities at 72 h under different conditions. Detailed production kinetics was shown in Additional file 1: Fig. S2. Specific intracellular transfructosylating activities peaked at 72 h while intracellular and extracellular activities based on per unit of cultivation volume peaked either at 72 h or 96 h because of increasing cell biomass concentrations. The total transfructosylating activities peaked at 72 h or 96 h though the values at 72 h were not statistically different from the values at 96 h.

**Table 2:** Transfructosylating activities of intracellular, extracellular and total enzymes after 72 h of shake-flask cultivation with different levels of nitrogen and phosphorous

Total (g/L)		Salt equivalent (g/L)		Cells (g/L)	Transfructosylating activity			
N	P	NaNO_3_	Na_2_HPO_4_		Intracellular (U/mg)	Intracellular (U/mL)	Extracellular (U/mL)	Total (U/mL)
0.92^a^	0.40^b^	5.6	1.8	6.7 ± 0.6	3.2 ± 0.0	21.4 ± 2.3	25.6 ± 1.7	47.1 ± 4.0
0.92^a^	0.65	5.6	3.0	8.2 ± 1.2	2.9 ± 0.0	23.6 ± 1.9	32.5 ± 1.4	56.1 ± 3.3
0.92^a^	0.87	5.6	4.0	8.7 ± 0.2	2.4 ± 0.0	20.9 ± 0.3	37.8 ± 1.5	58.7 ± 1.8
1.65	0.40	10.0	1.8	14.7 ± 0.9	4.6 ± 0.2	67.0 ± 0.7	56.8 ± 1.9	123.8 ± 2.6
1.65	0.65	10.0	3.0	15.3 ± 0.6	3.6 ± 0.0	55.8 ± 0.8	57.8 ± 1.8	113.6 ± 2.6
1.65	0.87	10.0	4.0	16.5 ± 0.2	3.4 ± 0.1	55.7 ± 2.5	59.5 ± 1.4	115.2 ± 3.9
2.48	0.40	15.0	1.8	13.7 ± 0.1	4.1 ± 0.1	56.2 ± 0.4	54.3 ± 5.7	110.6 ± 6.1
2.48	0.65	15.0	3.0	15.4 ± 0.5	3.6 ± 0.1	54.8 ± 1.1	55.4 ± 1.1	110.3 ± 2.2
2.48	0.87	15.0	4.0	16.2 ± 1.2	3.3 ± 0.0	53.9 ± 1.1	55.9 ± 3.3	109.8 ± 4.4

^a^Exogenous nitrogen was not added

^b^Exogenous phosphorous was not added

For specific intracellular activity (U/mg), it appeared that high phosphorous level had a negative effect and the highest intracellular activity at the same nitrogen level was achieved without addition of exogenous phosphorous. For example, the highest intracellular activity of 4.6 U/mg was achieved at 72 h with a total nitrogen concentration of 1.65 g/L (10.0 g/L NaNO_3_ equivalent) with addition of only 4.4 g/L NaNO_3_ (mid-N, low P). The second highest (4.1 U/mg) was achieved with a total nitrogen of 2.48 g/L (15.0 g/L NaNO_3_ equivalent) with addition of 9.4 g/L NaNO_3_ (high N, low P). Table 2 summarizes 72-h activity results. The highest intracellular transfructosylating activity of 67 U/mL based on per unit of cultivation volume was achieved with the addition of only 4.4 g/L NaNO_3_. The extracellular activity at this condition was at the similar level to other results with medium and high levels of nitrogen. The highest total activity of 123.8 U/mL was achieved with the addition of only 4.4 g/L (mid-N, low P), significantly higher than others (*p* < 0.5).

A few studies investigated the effect of nitrogen source on transfructosylating enzyme production using synthetic sucrose with various microorganisms including *Aspergillus* and *Aureobasidium* (Hayashi et al. 1992; Nascimento et al. 2019; Vandáková et al. 2004). These studies often led to different conclusions on the best nitrogen source, possibly depending on the strains and other cultivation conditions. Moreover, relatively high concentrations of nitrogen sources (10–20 g/L) and phosphorous sources (~ 5 g/L) together with other nutrients such as magnesium are often used in synthetic sucrose media for producing transfructosylating enzymes (Aung et al. 2019; Dominguez et al. 2012; Jung et al. 2011; Salinas and Perotti 2009; Sangeetha et al. 2004a, b; Shin et al. 2004; Vandáková et al. 2004; Zhang et al. 2019). In the studies of transfructosylating enzyme production by *Aureobasidium* strains, combination of yeast extract with NaNO_3_ is widely used in synthetic sucrose medium (Aung et al. 2019; Jung et al. 2011; Salinas and Perotti 2009; Vandáková et al. 2004; Yoshikawa et al. 2006; Zhang et al. 2019). Some studies reported the production of transfructosylating enzymes using molasses by *Aspergillus* strains as media but high-cost organic nitrogen sources such as yeast extract and peptone were still used at relatively high concentrations (e.g., 15–30 g/L) with others (Dorta et al. 2006; Xie et al. 2017). The present study, however, showed that addition of yeast extract and phosphorous, and over-dosage of NaNO_3_ only increased cell biomass production but did not improve enzyme production. Since molasses already contained nitrogen (a significant portion is derived from sugarcane proteins), phosphorous and other nutrients, only low-cost inorganic nitrogen was required at a relatively low concentration. Over-dosage of nitrogen and phosphorous may lead to the rapid production of cell growth and suppresses the expression of transfructosylating enzymes.

### Transfructosylating enzyme production in 1 L bioreactor

**Fig. 3:**
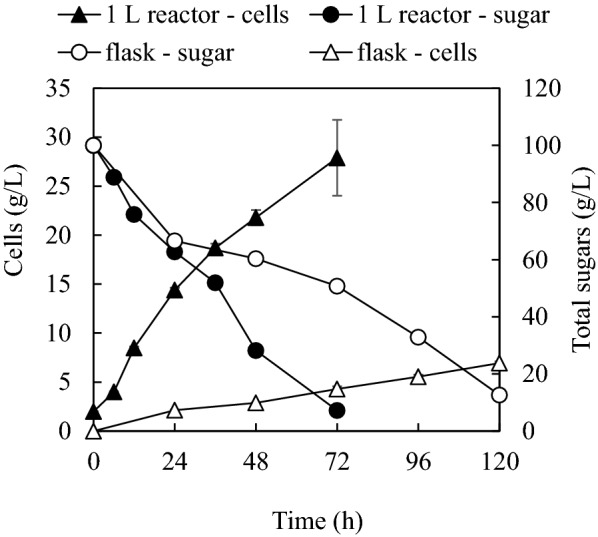
Comparison of cell growth and sugar consumption in shake flasks and 1 L bioreactor. Total nitrogen, 1.65 g/L and equivalent to 10 g/L NaNO_3_ (with addition of only 4.4 g/L exogenous NaNO_3_); total phosphorous, 0.40 g/L and equivalent to 1.8 g/L Na_2_HPO_4_ (no exogenous phosphorous)

Transfructosylating enzyme production was scaled to 1 L bioreactor using molasses medium with addition of only 4.4 g/L exogenous NaNO_3_. Figure 3 shows and compares cell biomass and sugar consumption at shake flaks and the reactor scale. Cultivation in 1 L bioreactor significantly improved cell growth rate and sugar consumption. After 72-h cultivation, only 7.2 g/L residual sugars were left, and cell biomass concentration reached 27.9 g/L compared to residual sugars of 12.6 g/L and cells of 23.8 g/L after 120-h cultivation in shake flask.

**Fig. 4:**
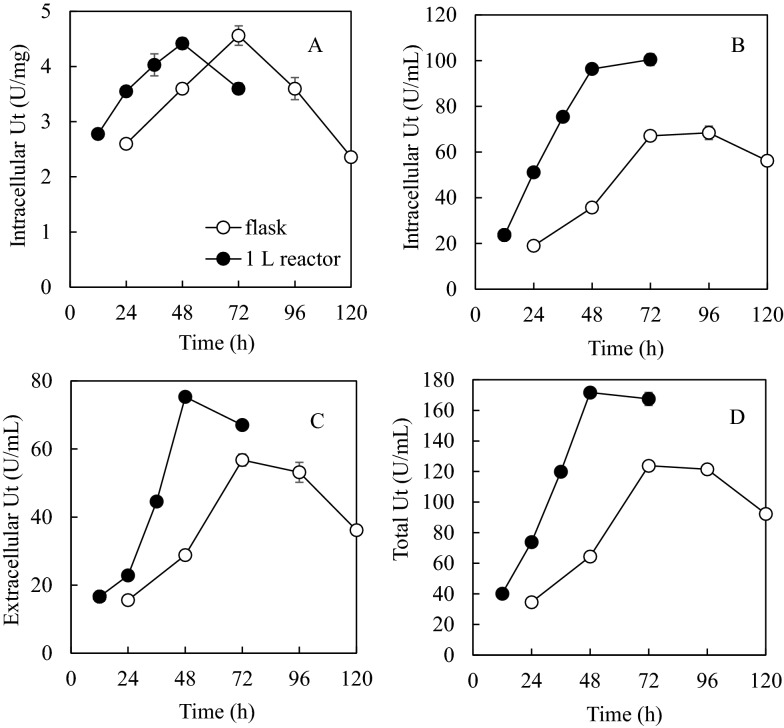
Comparison of specific intracellular transfructosylating activity (**A**), and intracellular (**B**), extracellular (**C**) and total (**D**) enzyme activities based on per unit of volume in shake flasks and 1 L reactor. Nutrient conditions shown in Fig. 3

Figure 4 compared enzyme production in shake flask and bioreactor. For intracellular enzymes, the maximum specific activity of 4.4 U/mg in the reactor was achieved at only 48 h cultivation compared to 4.6 U/mg after 72 h cultivation in shake flask. The intracellular activity based on per unit of volume reached a platform after 48 h as cell concentration increase offset the specific activity drop. The intracellular, extracellular, and total activities at 48 h reached 96.4 U/mL, 75.3 U/mL, and 171.7 U/mL, 44%, 33% and 39% higher than those corresponding activities achieved at 72 h in shake flasks, respectively. The productivity of total activity was 3.58 U/mL/h, 108% higher than that achieved in shake flasks.

The improved production of cell biomass and enzymes, and the rapid consumption of sugars were likely attributed to the improved mixing, mass transfer and oxygen supply. In the 1 L reactor, the agitation speed was in the range of 350 to 500 rpm and the dissolved oxygen level was controlled at above 20% by adjusting agitation speed and aeration rate (0.5–0.9 vvm). In contrast, flask cultivation was conducted in an orbital shaking incubator at 180 rpm.

Table 3 compares the transfructosylating activities obtained in the present study with the results reported in literature by *Aureobasidium* strains. The enzyme activity achieved in the present study in the 1 L reactor is lower than those (550–2100 U/mL) achieved with some engineered strains of *Aureobasidium* (Zhang et al. 2019). However, this value is much higher than many other studies using wild strains using either sucrose or molasses medium (Shin et al. 2004; Vandáková et al. 2004). The results from the present study also indicate that production of transfructosylating enzymes from sugarcane molasses by *A. pullulans* FRR 5284 can be further improved through process optimization and strain engineering.

**Table 3:** Comparison of transfructosylating activities produced by *Aureobasidium* strains

*Aureobasidium* strain	Sucrose (g/L)	Exogenous nutrients—NaNO_3_, yeast extract, phosphate salts (g/L)	Shake flask/reactor	Extraction (yes/no)	Transfructosylating activity (U/mL)			References
					Intra-	Extra-	Total	
*A. pullulans* FRR 5284	100 (molasses sugars)	4.4, 0.0, 0.0	Shake flask	No	67.0	56.8	124	This study
*A. pullulans* FRR 5284	100 (molasses sugars)	4.4, 0.0, 0.0	1 L reactor	No	96.4	75.2	172	This study
*A. pullulans* FRR 5284	100	10.0, 15.0, 5.5^b^	Shake flask	No	38.7	–	115	Khatun et al. (2020)
*A. pullulans* KCCM 12,017	100	10.0, 15.0, 5.5^b^	Shake flask	No	67	54	121	Shin et al. (2004)
*A. pullulans* DSM 2404	50	10.0, 20.0, 5.0^b^	Shake flask	Yes	47.3	–	–	Yoshikawa et al. (2006)
*A. pullulans* CCY 27-1-94	200	10.0, 10.0, 5.0^b^	Shake flask	No	60	50	110	Vandáková et al. (2004)
*Aureobasidium* sp. ATCC 20524	200	10.0, 20.0, 5.0^b^	Shake flask	No	–	–	103	Hayashi et al. (1990)
*A. melanogenum* 33	180	15.0, 20.0, 5.0^c^	Shake flask	Yes	218.7	–	–	Aung et al. (2019)
*A. melanogenum* D28^a^	180	15.0, 20.0, 5.0^c^	10-L reactor	Yes	2100	–	–	Zhang et al. (2019)

^a^*A. melanogenum* is the mutant of *A. melanogenum* 33

^b^K_2_HPO_4_

^c^KH_2_PO_4_

### Comparison of FOS production using intracellular, extracellular, and total enzymes from shake flasks and reactor

FOS production was compared using intracellular, extracellular, and total enzymes from both shake flasks (72 h) and the reactor (48 h) at the activity loading of 20 U/mL reaction solution. Table 4 summarizes the FOS yields by using either the individual or mixed enzymes from shake flasks and the reactor. The FOS yield results show that FOS production was rapid and the yields with most enzyme samples reached 60% or more after only 3-h reaction. The total FOS yields reduced, which was attributed to the further hydrolysis of FOS to individual reducing sugars.

**Table 4:** FOS yields using intracellular, extracellular and mixed enzymes from shake flasks and 1 L reactor

Batch	Enzyme type	FOS yield (%)			
		1 h	3 h	6 h	12 h
Shake flask	Intracellular—cells	58.0 ± 2.0	61.2 ± 0.6	62.0 ± 0.1	54.0 ± 0.5
	Extracellular—broth	55.2 ± 1.0	58.4 ± 0.2	60. 2 ± 1.2	56.0 ± 0.9
	Mixed	56.3 ± 1.1	60.1 ± 0.5	61.5 ± 1.0	53.7 ± 0.9
1 L bioreactor	Intracellular—cells	56.3 ± 1.1	60.1 ± 0.5	61.5 ± 1.0	56.5 ± 0.8
	Extracellular—broth	59.2 ± 1.0	61.7 ± 0.5	62.7 ± 0.2	58.2 ± 0.5
	Mixed	57.7 ± 0.6	61.3 ± 0.7	61.4 ± 0.2	57.4 ± 0.6

**Fig. 5:**
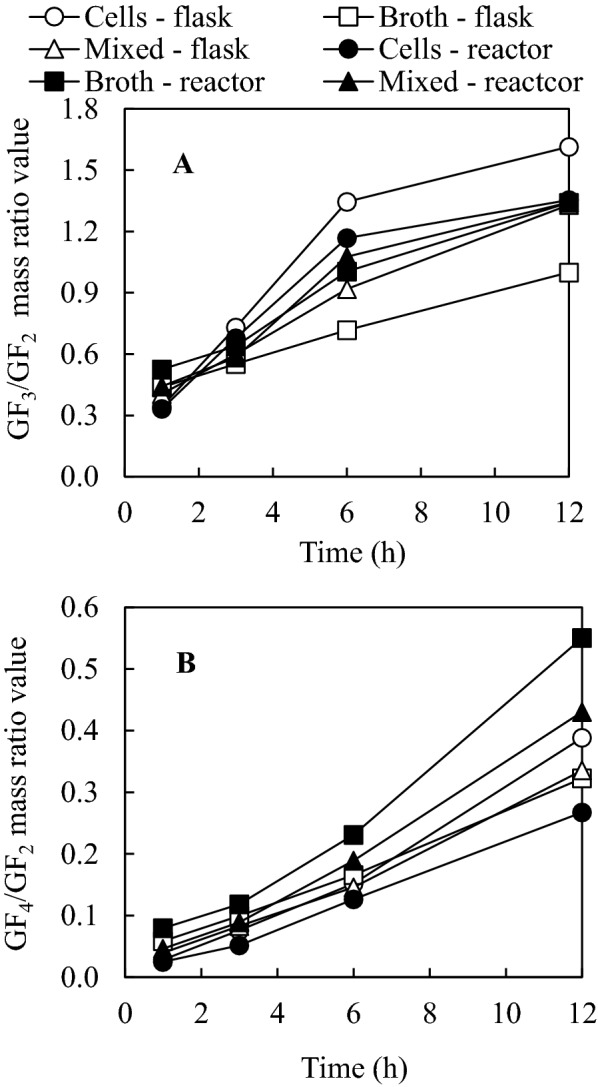
Comparison of GF_3_/GF_2_ and GF_4_/GF_2_ ratio values using intracellular, extracellular and total enzymes from shake flasks and 1 L bioreactor. Reaction conditions, 20 U/mL solution, 500 g/L sucrose, 55 °C and pH 5.5

The prebiotic activity of FOS is also affected by the FOS composition (Nobre et al. 2018). Understanding the change of GF_2_/GF_3_/GF_4_ ratio will help to determine the reaction time to achieve the preferred ratio of GF_2_/GF_3_/GF_4_. In order to track the ratio changes with different enzymes, relative ratio changes of GF_3_ and GF_4_ compared to GF_2_ (as 1.0) were monitored. Additional file 1: Figs. S3–S5 show some representative HPLC chromatographs of individual FOS by using reactor enzymes while Fig. 5 shows the ratios of GF3/GF2 and GF4/GF2 based on the integrated and calculated results from HPLC chromatographs. Interestingly, although the total FOS yields were in the similar levels (Table 4), the ratios of GF_3_ and GF_4_ compared to GF_2_ varied using different samples. The use of intracellular enzymes from shake flasks led to the highest GF_3_/GF_2_ ratios after 3-h reaction while the use of intracellular enzymes resulted in the lowest GF_3_/GF_2_ ratio. In contrast, the GF_3_/GF_2_ ratio difference between using intracellular and extracellular enzymes from the reactor was in a much smaller range. Regarding the GF_4_/GF_2_ ratio, the use of extracellular enzymes from the reactor led to the highest GF_4_/GF_2_ ratios through the reaction while the use of reactor intracellular enzymes had the least GF_4_/GF_2_ ratios. In contrast to GF_3_/GF_2_ ratio, the difference between the GF_4_/GF_2_ ratios using intracellular and extracellular enzymes from shake flasks was in a much small range. Figure 5 also shows that the use of the mixed enzymes containing both intracellular and extracellular enzymes led to the GF_3_/GF_2_ and GF_4_/GF_2_ ratios in between those using the individual intracellular and extracellular enzymes. To the authors’ knowledge, comparison of FOS profiles using intracellular and extracellular transfructosylating enzymes has not been previously reported.

Previously, it was found that FOS from *Aspergillus ibericus* with GF_2_/GF_3_/GF_4_ ratio of 1:1.28:0.10 had higher activities in promoting the growth of a number of probiotics in an in vitro digestion study compared to FOS from *A. pullulans* CCY 27-1-94 with GF_2_/GF_3_/GF_4_ ratio of 1:1.61:0.21 (Nobre et al. 2018). Moreover, there are a few commercial FOS products with different FOS composition. For example, Profeed (Tereos, France) and Nutraflora P-95 (Ingredion, Canada) have similar GF_3_/GF_2_ ratios of 1.43:1 but different GF_4_/GF_2_ ratios (0.27:1 for Profeed vs. 0.36:1 for Nutraflora) according to their product specifications. In contrast, Actilight (Tereos, France) and one FOS product from GCT Nutrition had a similar high level of GF_3_/GF_2_ ratio of ~ 1.7:1 but different GF_4_/GF_2_ ratios (0.20:1 for Actilight and 0.30:1 for GCT FOS) (Kaplan and Hutkins 2000; Nobre et al. 2019). Studies also showed probiotics such as *Lactobacillus* and *Bifidobacterium* strains had different responses towards FOS products such as NutraFlora P-95, Raftilose P95 and Inulin-S from different companies and/or sources (Huebner et al. 2007). The results from the present study indicated that FOS profiles could be controlled through combination of the activity ratio of intracellular and extracellular enzymes and control of reaction time for various applications. This will be very useful to produce FOS with different GF_2_/GF_3_/GF_4_ ratios for applications.

## Conclusions

In this study, sugarcane molasses was used as a low-cost carbon source for the production of transfructosylating enzymes by a newly identified *A. pullulans* strain. Relatively high transfructosylating activity was achieved with only the addition of 4.4 g/L NaNO_3_ into molasses-based medium. Moreover, transfructosylating production using molasses-based medium supplemented with NaNO_3_ was demonstrated at a 1 L bioreactor with significant improvements in activity and productivity. This study also demonstrated that FOS composition could be controlled by selecting specific the activity ratio of intracellular and extracellular enzymes and/or reaction time, which is useful for the production of FOS different compositions for food and feed applications.

### Supplementary Information

**Additional file 1.** **Experiment method:** Elemental analysis of molasses. **Fig. S1.** Effect of exogeneous nitrogen and phosphorous on cell growth (A) and sugar consumption (B). Total levels of N and P shown in Table 1. **Fig. S2.** Effect of exogeneous nitrogen and phosphorous on specific intracellular transfructosylating activity (A), intracellular (B), extracellular (C), and total (D) enzyme activities based on per unit of volume. Total levels of N and P shown in Table 2. **Fig. S3.** HPLC chromatographs of FOS samples from 1 h enzymatic hydrolysis using reactor enzymes. **Fig. S4.** HPLC chromatographs of FOS samples from 3 h enzymatic hydrolysis using reactor enzymes. **Fig. S5.** HPLC chromatographs of FOS samples from 12 h enzymatic hydrolysis using reactor enzymes

## Data Availability

The data and the materials are all available in this article and Additional file.

## Abbreviations

*Aureobasidium pullulans*: *A. pullulans*
FOS: Fructooligosaccharides
GF_2_: Kestose
GF_3_: Nystose
GF_4_: 1,1,1-Kestopentaose
U_t_: Transfructosylating activity
U_h_: Hydrolysis activity
