# Supplementary material for: Highly efficient production of transfructosylating enzymes using low-cost sugarcane molasses by A. pullulans FRR 5284
Source: Bioresour Bioprocess. 2021 Jun 11;8(1):48. doi: 10.1186/s40643-021-00399-x (PMC10992317; doi:10.1186/s40643-021-00399-x)
Supplement: Supplementary file 1 — Additional file 1. Experiment method: Elemental analysis of molasses. Fig. S1. Effect of exogeneous nitrogen and phosphorous on cell growth (A) and sugar consumption (B). Total levels of N and P shown in Table 1. Fig. S2. Effect of exogeneous nitrogen and phosphorous on specific intracellular transfructosylating activity (A), intracellular (B), extracellular (C), and total (D) enzyme activities based on per unit of volume. Total levels of N and P shown in Table 2. Fig. S3. HPLC chromatographs of FOS samples from 1 h enzymatic hydrolysis using reactor enzymes. Fig. S4. HPLC chromatographs of FOS samples from 3 h enzymatic hydrolysis using reactor enzymes. Fig. S5. HPLC chromatographs of FOS samples from 12 h enzymatic hydrolysis using reactor enzymes [file 40643_2021_399_MOESM1_ESM.docx]

**Highly efficient production of transfructosylating enzymes using low-cost sugarcane molasses by *A. pullulans* FRR 5284**

Most Sheauly Khatun ^1,2^, Morteza Hassanpour ^1,2^, Mark D. Harrison ^1,3^, Robert E. Speight ^1,3^, Ian M. O’Hara ^1,2^, Zhanying Zhang ^1,2*^

^1^Centre for Agriculture and the Bioeconomy, Faculty of Science, Queensland University of Technology, Brisbane, QLD 4000, Australia.

^2^School of Mechanical, Medical and Process Engineering, Faculty of Engineering, Queensland University of Technology, Brisbane, QLD 4000, Australia.

^3^School of Biology and Environmental Science, Faculty of Science, Queensland University of Technology, Brisbane, QLD 4000, Australia.

^*^ Corresponding author, Dr Zhanying Zhang

Email: [jan.zhang@qut.edu.au](mailto:jan.zhang@qut.edu.au)

## **Elemental analysis of molasses**

The total nitrogen was determined by TOC/TN analyser (Shimadzu, Japan) coupled with chemi-luminescence detector (CLD). The diluted molasses (100 time) samples were placed in a combustion chamber using air, all nitrogen converted NO at 720 ℃ via catalytic oxidation. The gas containing NO moved in chemi-luminescence detector (CLD) to determine the concentration of NO.

For the measurement of mineral concentration, molasses samples were diluted 100 times in ultra-pure milli-Q water. 9.8mL was aliquoted into separate test tubes and then 200µL of 70% nitric acid (Thermo Fisher Scientific) was added. Each sample was processed in triplicate for mineral analysis. The concentrations of minerals were determined using inductively coupled plasma optical emission spectrometry (ICP-OES) on a Perkin Elmer ICP-OES 8300DV fitted with an ESI SC-4DX autosampler and prepFAST 2 sample-handling unit for online internal standardisation and auto-dilution of calibration standards purified nitric acid was used for the preparation of all standards and blank solutions. Instrument calibration was performed using multi-element standards (High Purity Standards, Charleston, USA). Samples were analysed using an integration time of 0.15 s with three replications, at the following wavelengths K (766.490–Rad), Mg (285.213-Rad), P (213.617-Rad), Ca (317.933-Rad), and Fe (259.939-Rad). Concentrations were expressed in ppm.


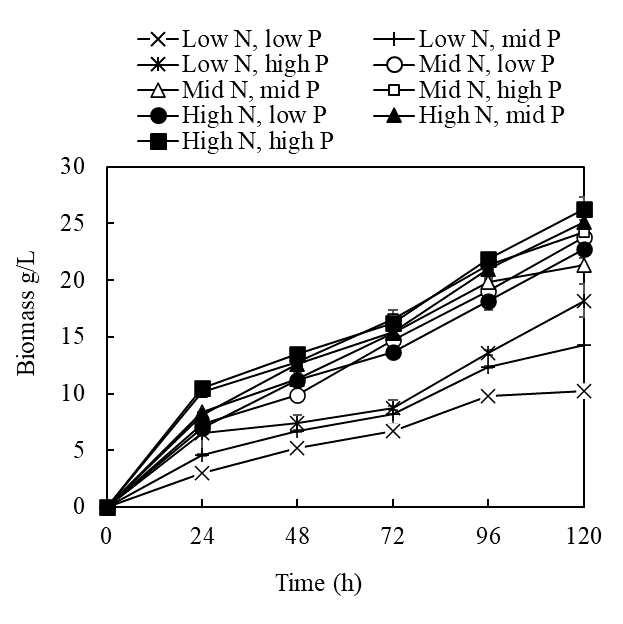

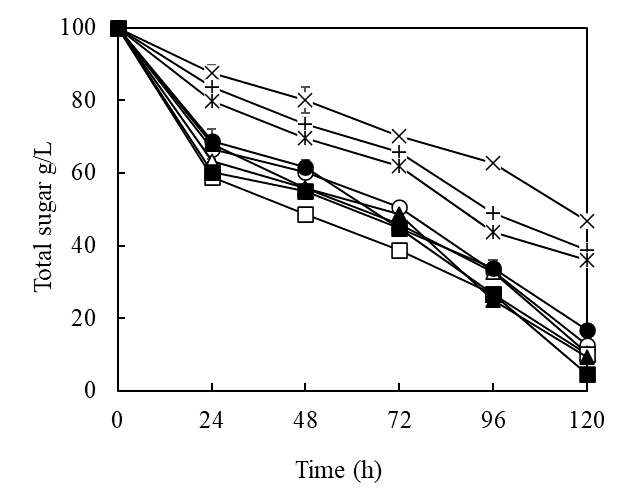


B

A

**Fig. S1**. Effect of exogeneous nitrogen and phosphorous on cell growth (A) and sugar consumption (B). Total levels of N and P shown in Table 1.


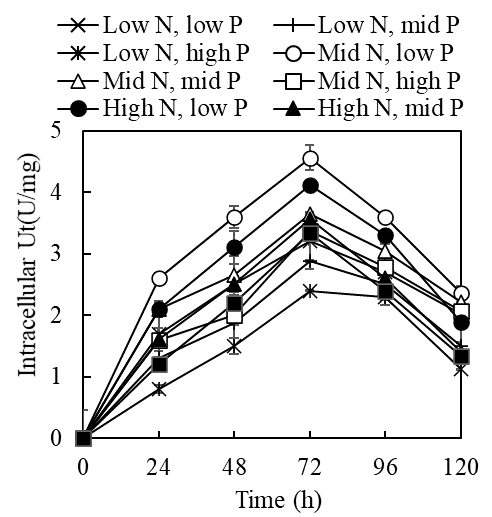

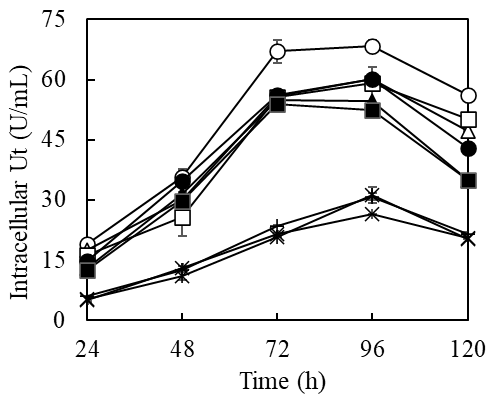

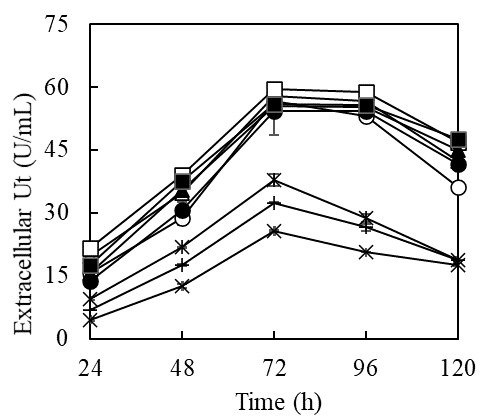


A

C

D

B


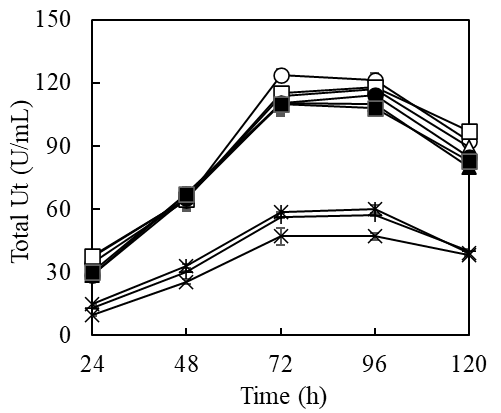


**Fig. S2.** Effect of exogeneous nitrogen and phosphorous on specific intracellular transfructosylating activity (A), intracellular (B), extracellular (C), and total (D) enzyme activities based on per unit of volume. Total levels of N and P shown in Table 2.


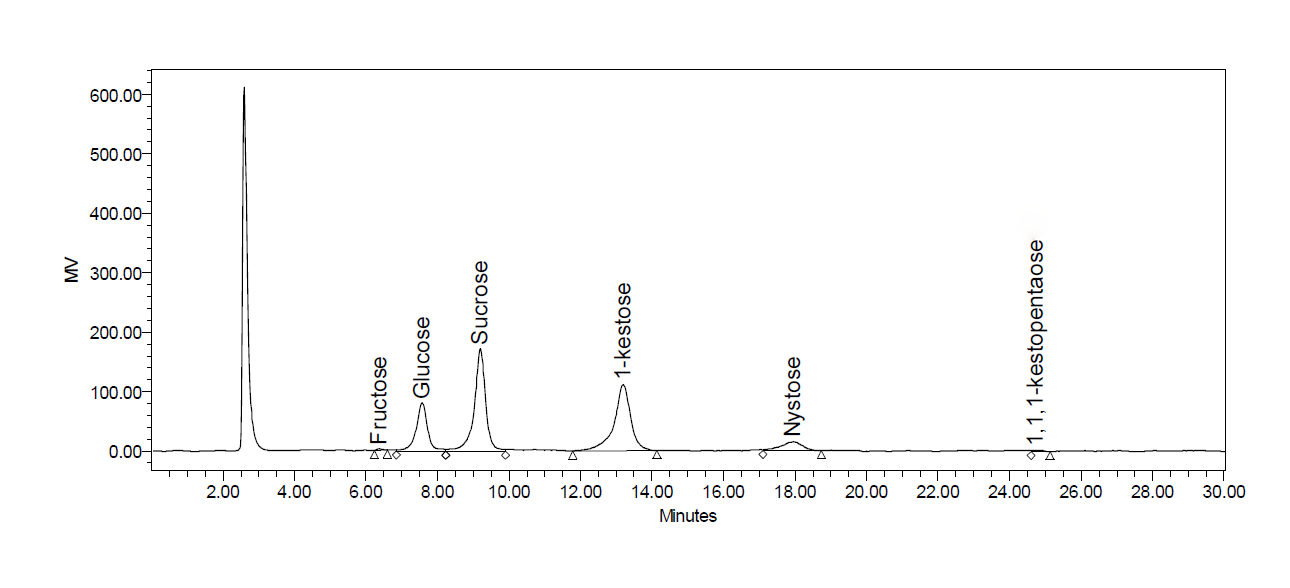

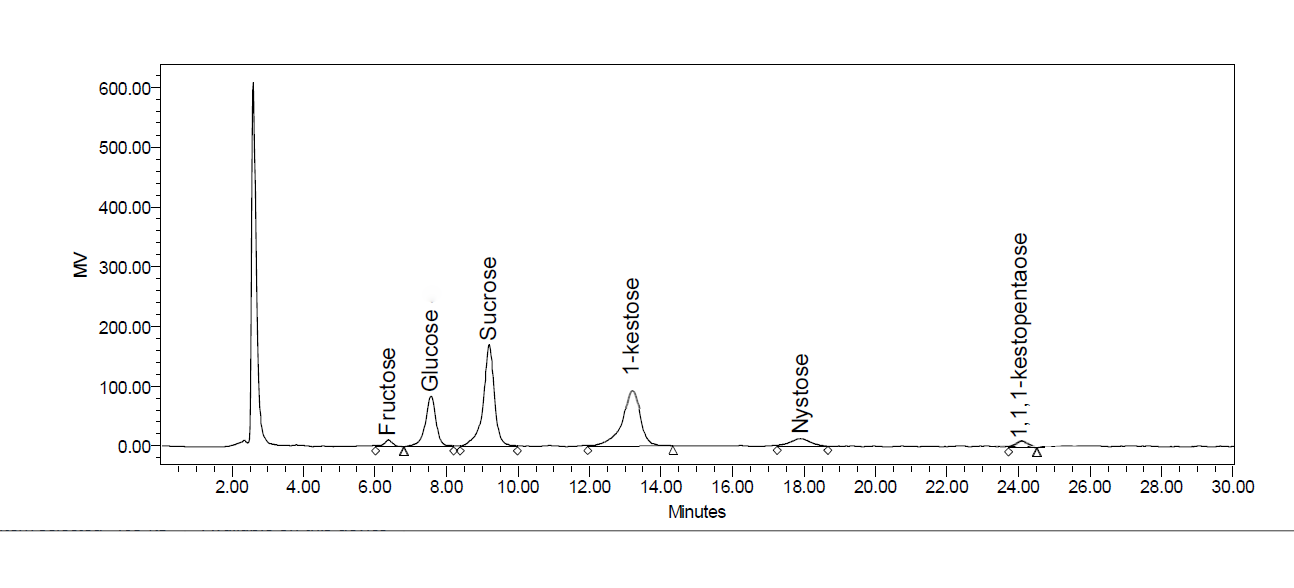

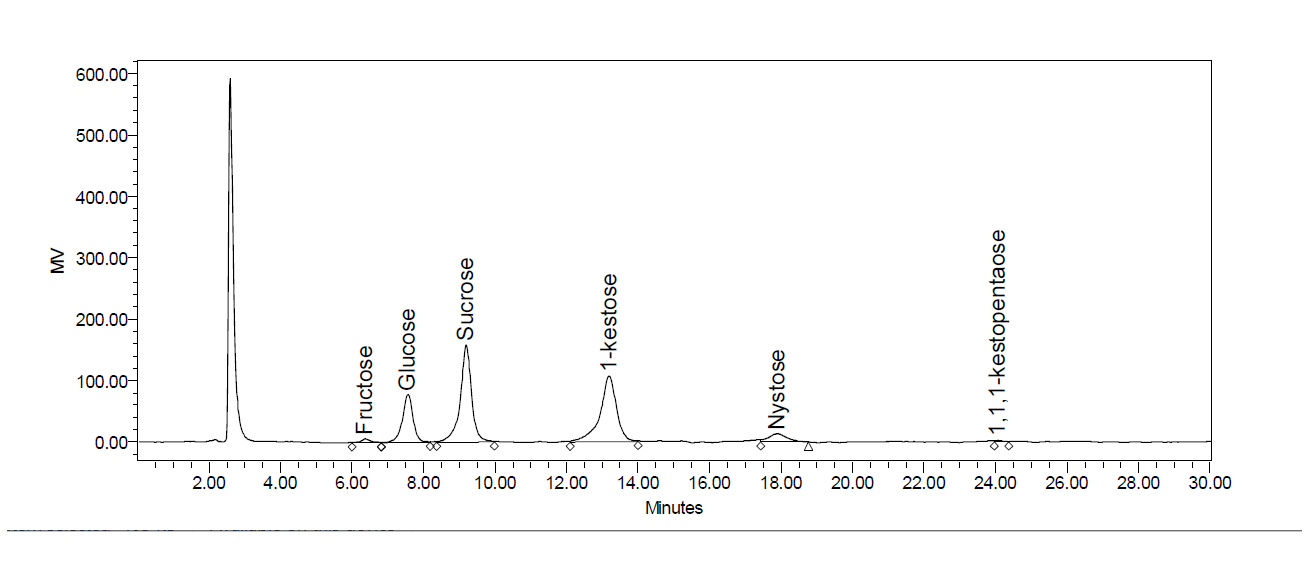


Cells-1 h

Broth-1 h

Mixed-1 h

**Fig. S3.** HPLC chromatographs of FOS samples from 1 h enzymatic hydrolysis using reactor enzymes


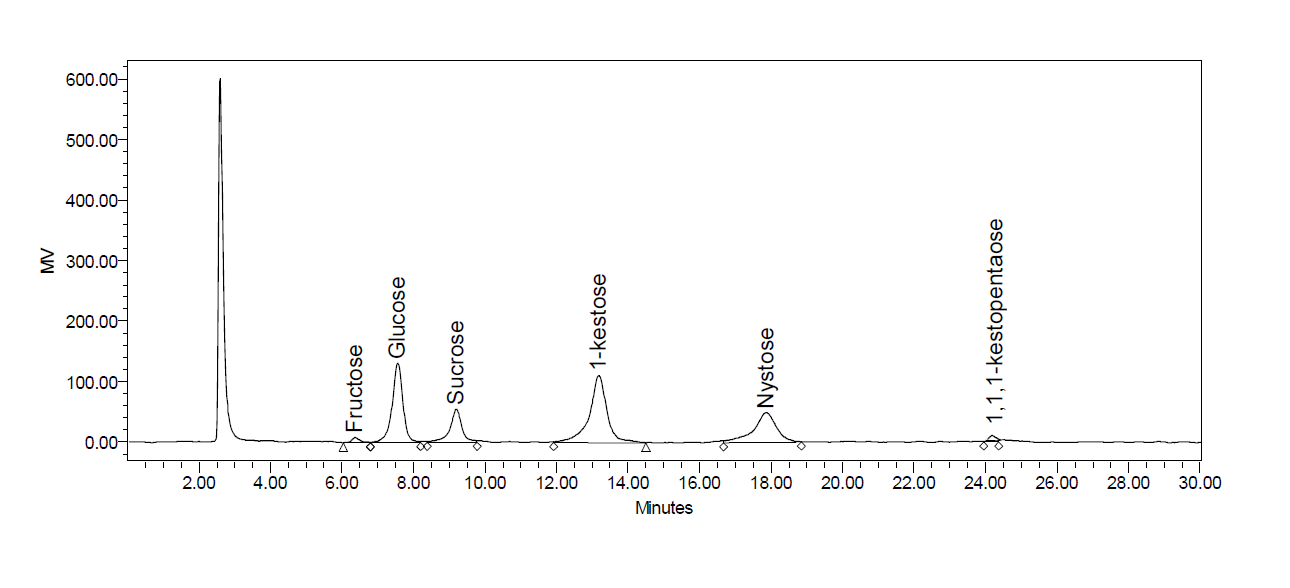

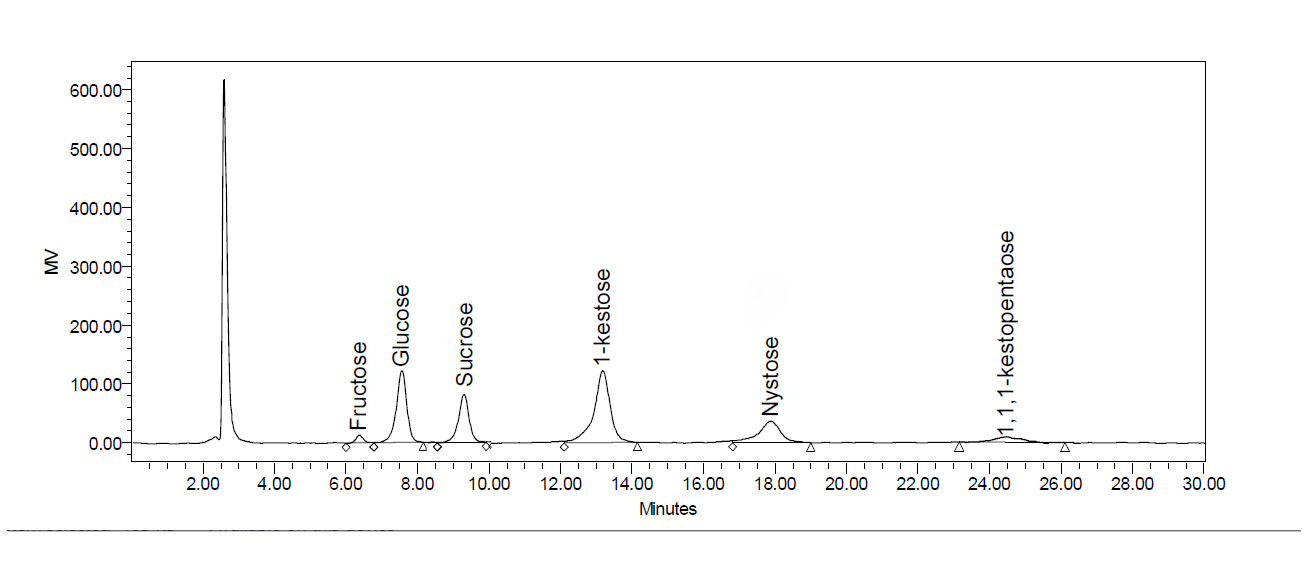

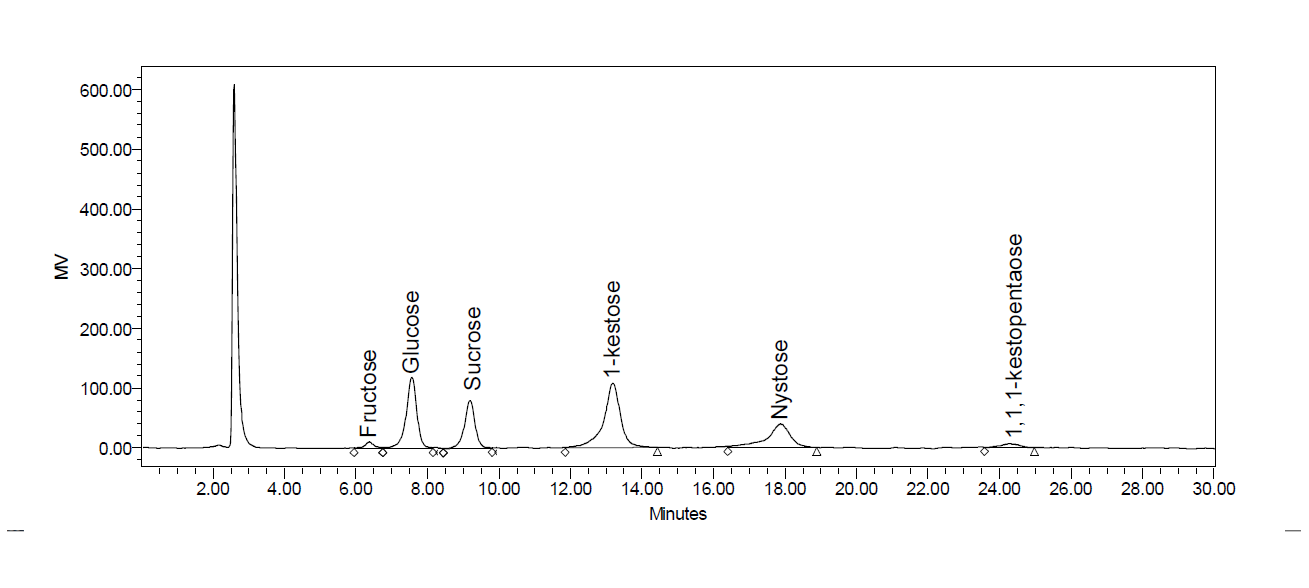


Cells-3 h

Broth-3 h

Mixed-3 h

**Fig. S4.** HPLC chromatographs of FOS samples from 3 h enzymatic hydrolysis using reactor enzymes


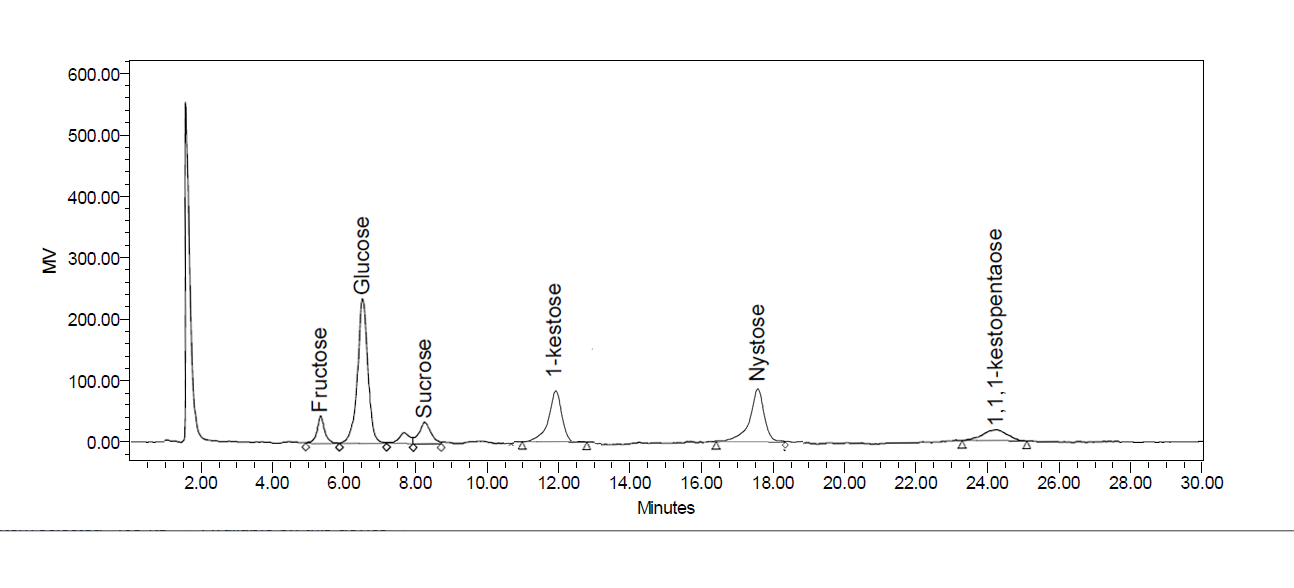

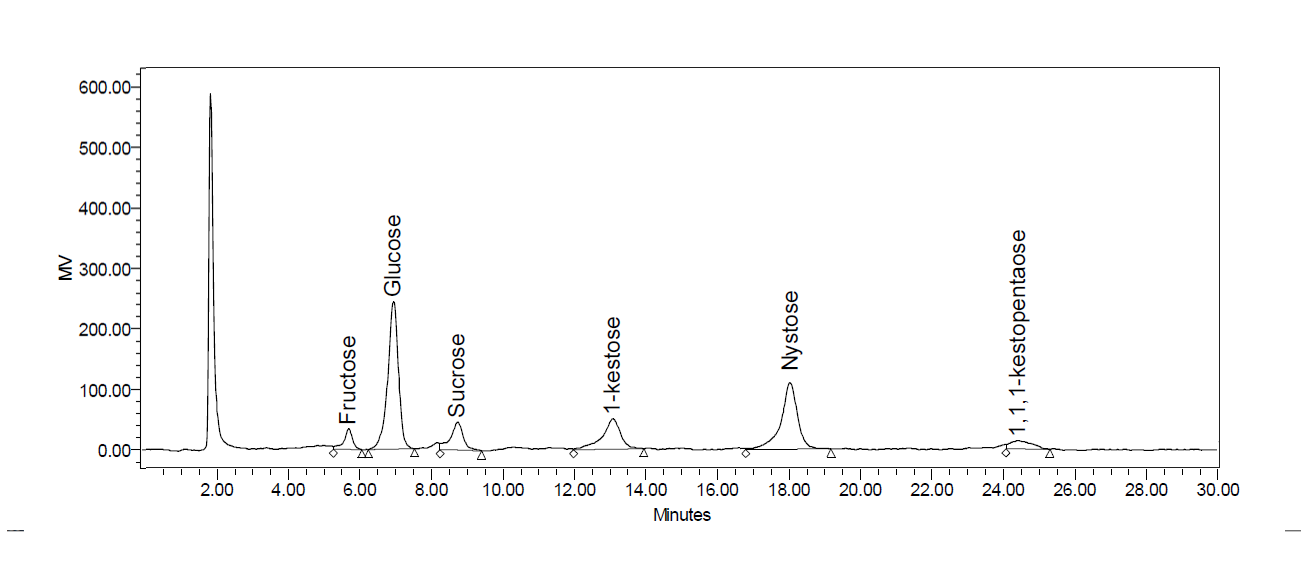

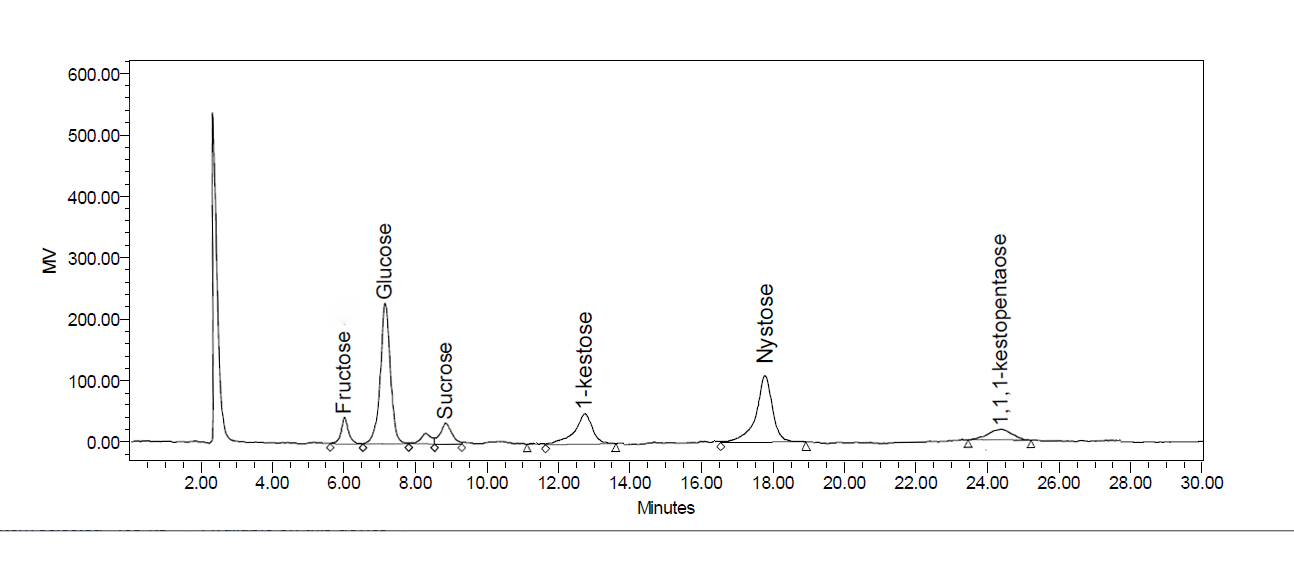


Cells-12 h

Broth-12 h

Mixed-12 h

**Fig. S3.** HPLC spectra of FOS samples from 3 h enzymatic hydrolysis

**Fig. S5.** HPLC chromatographs of FOS samples from 12 h enzymatic hydrolysis using reactor enzymes
